# The relationship between characteristics of gait disturbance and injury patterns of the corticospinal tract and corticoreticular pathway in post-stroke patients: A case series of 3 patients

**DOI:** 10.1097/MD.0000000000034195

**Published:** 2023-07-28

**Authors:** Ren Fujii, Makoto Tamari, Naomichi Mizuta, Naruhito Hasui, Yuki Nonaka, Fumiaki Tamiya, Misato Horinouchi, Hiroshi Hosokawa, Shinichiro Tanaka

**Affiliations:** a Musashigaoka Clinical Research Center, Musashigaoka Hospital (Tanakakai Medical Corp.), Kumamoto, Japan; b Department of Rehabilitation, Musashigaoka Hospital (Tanakakai Medical Corp.), Kumamoto, Japan; c Department of Physical Therapy, Faculty of Rehabilitation, Reiwa Health Science University, Fukuoka, Japan; d Department of Rehabilitation, Faculty of Health Sciences, Nihon Fukushi University, Aichi, Japan; e Depertment of Therapy, Takarazuka Rehabilitation Hospital (Showakai Medical Corp.), Hyogo, Japan; f Department of Rehabilitation Medicine, Musashigaoka Hospital (Tanakakai Medical Corp.), Kumamoto, Japan.

**Keywords:** abnormal gait pattern, corticoreticular pathway, corticospinal tract, gait disturbance, post-stroke patient

## Abstract

Corticospinal tract (CST) and corticoreticular pathway (CRP) injury patterns (i.e., the continuity of the nerve fibers) are associated with gait disturbance in post-stroke patients. In this case series study, we examined the case of 3 patients with different CST and CRP injury patterns and analyzed the characteristics of gait disturbance in each patient.

**Patient concerns:**

Patient 1 (P1) was a 73-year-old woman who presented with paralysis of the right upper and lower extremities due to a left lacunar infarction. Patient 2 (P2) was a 41-year-old man who presented with paralysis of the right upper and lower extremities due to a left putamen hemorrhage. Patient 3 (P3) was a 57-year-old man who presented with paralysis of the left upper and lower extremities due to a right putamen hemorrhage.

**Diagnosis:**

In P1, the CRP in the affected hemisphere was intact, but the CST was discontinuous. In P2, the CST in the affected hemisphere was intact, but the CRP was discontinuous. P3 was discontinuous in both CST and CRP in the affected hemisphere.

**Outcomes:**

Over time, all 3 patients improved to the level of gait independence, but they exhibited different gait patterns. Among them, P3 showed a markedly abnormal gait pattern that included spatiotemporal gait asymmetry, lateral shift of the trunk, and hip hiking.

**Lessons:**

This case series study demonstrated that even if both the CST and CRP were injured, gait recovered to some extent (i.e., independent level-ground gait), but the abnormal gait pattern might remain remarkable.

## 1. Introduction

The importance of rehabilitation designed for the improvement of gait disturbances has been widely recognized.^[1]^ Gait disturbance is associated with various factors, including motor paralysis, sensory disturbance, muscle weakness on the nonparalyzed side, trunk impairment, cognitive dysfunction, and higher-brain dysfunction.^[2–7]^ Neurological factors also influence gait disturbance, and among these factors, it is important to evaluate of the degree of injury to the corticospinal tract (CST) and the corticoreticular pathway (CRP) as visualized by diffusion tensor tractography (which is derived from diffusion tensor imaging [DTI]) in order to understand the pathology of gait disturbance.^[8–10]^

The CST is the major neuronal pathway that mediates voluntary movements.^[11]^ The main function of the CST is control of the distal musculature used for fine movement.^[11]^ In contrast, the CRP mediates mainly the proximal and trunk muscles to control posture and gait.^[11]^ Several studies have demonstrated the usefulness of diffusion tensor tractography examinations of the CST and CRP for the prediction of gait ability in individuals who have experienced a stroke.^[8–13]^ Yoo et al^[10]^ investigated CST and CRP injury patterns and reported that patients whose CST and CRP were both injured had significantly decreased gait performance. It is thus clear that injuries to the CST and CRP influences gait independence, but it is not yet clear how damage to these neural pathways is involved in abnormal gait patterns.

Although both the CST and CRP are involved in gait performance, each neural pathway has a different role in gait control.^[11]^ We speculate that the characteristics of abnormal gait patterns may therefore differ depending on the injury patterns of the CST and CRP. It is important to identify factors that are associated with abnormal gait patterns, because most patients with stroke wish to improve their gait pattern. In this case series study, we analyzed the case of 3 patients with different CST and CRP injury patterns and identified the characteristics of the abnormal gait pattern in each patient.

## 2. Case presentation

This case series was comprised of 3 patients who were diagnosed with cerebral infarction or cerebral hemorrhage and underwent rehabilitation at Musashigaoka hospital between May 2021 and November 2021. All procedures were approved by the ethics committee of Musashigaoka Hospital of Medical Corporation Tanakakai (approval no. R03-01) and were conducted in accord with the tenets of the Declaration of Helsinki. Patient data including sex, age, symptoms, progress, and inspection results were retrieved and retrospectively reviewed. Informed written consent was obtained from all patients and their families.

The patient 1 (P1) was a 73-year-old right-handed woman who presented with severe paralysis of the right upper and lower extremities due to a left lacunar infarction. T2-weighted MRI images showed a lesion in the left deep white matter (left putamen and corona radiata) at 4 weeks after the infarction’s onset. After treatment at an acute care hospital, the patient was admitted to our hospital for comprehensive rehabilitation 10 days after the stroke onset (Table 1).

**Table 1:** Patient demographic data.

Patient no.	P1	P2	P3
Sex/age, yr	Female/73	Male/41	Male/57
Days post-stroke, d	10	44	71
Length of stay, d	96	101	109
Stroke type	Infarct	Hemorrhage	Hemorrhage
Lesion side	Left	Left	Right
Type of injury	Group B	Group C	Group D

Group A: patients with intact CST and CRP, Group B: patients with injured CST and intact CRP, Group C: patients with intact CST and injured CRP, Group D: patients with injured CST and CRP.

CRP = corticoreticular pathway, CST = corticospinal tract.

Patient 2 (P2) was 41-year-old right-handed man who presented with severe paralysis of the right upper and lower extremities due to a left putamen hemorrhage. T2-weighted MRI images showed a hematoma in the left putamen at 8 weeks after the stroke’s onset. After treatment at an acute care hospital, P2 was admitted to our hospital for comprehensive rehabilitation 6 weeks after the stroke onset (Table 1).

Patient 3 (P3) was 57-year-old right-handed man who presented with severe paralysis of the left upper and lower extremities due to a right putamen hemorrhage. T2-weighted MRI images showed a hematoma in the right putamen at 12 weeks post-onset. After treatment at an acute care hospital, P3 was admitted to our hospital for comprehensive rehabilitation 10 weeks after the stroke onset (Table 1).

### 2.1. Diffusion tensor tractography

DTI scanning was performed at the time of each patient’s admission, using a multi-channel head coil on a 1.5-T MR system (Echelon RX, Hitachi, Tokyo, Japan) with single-shot echo-planar imaging. We acquired 24 contiguous slices parallel to the anterior commissure-posterior commissure line in each patient. Scanning was performed from the cortex to the inferior surface of cerebellar hemisphere. Imaging parameters were as follows: TE = 90 ms; repetition time = 4900 ms; FA = 90°; slice thickness = 5 mm; FOV = 23.0 × 23.0 cm; recon matrix = 256 × 256; freq/phase = 96 × 96; voxel size = 2.395 × 2.395 × 5.0 mm; bandwidth = 250 kHz; b-value = 500 s/mm^2^; number of diffusion-encoding directions = 13. Three-dimensional reconstruction of fiber tracts was obtained using DSI Studio software (https://dsi-studio.labsolver.org/). Fiber tracking was conducted as described.^[14,15]^

In the CST, we first positioned a seed region of interest in the cerebral peduncle. According to the tracking fibers, we identified the fibers passing through the cerebral peduncle, crus posterus capsulae internae, and the radiate crown, and after that the fibers projecting to the central sulcus and the motor area. Using another region at the level immediately after the bifurcation of the motor and sensory cortex, the CST was identified. In the CRP, a seed region of interest was placed on the midbrain tegmentum. According to the tracking fibers, we comparted the CRP and the already-constructed CST pathways and identified fibers that projected to the premotor cortex. The FA threshold to terminate the tracking was 0.2°, and the angle ≥ 50°.

We identified the injury state of the CST and/or CRP in the affected hemisphere in the morphology of these fibers (i.e., discontinuation of a neural tract at sites around the hematoma). We then classified the 3 patients based on 4 groups according to the injury of the CST and/or CRP as follows according to the definition of Yoo et al^[10]^: Group A: patients with intact CST and CRP, Group B: patients with an injured CST and an intact CRP, Group C: patients with an intact CST and an injured CRP, and Group D: patients with an injured CST and an injured CRP.

**Figure 1.**
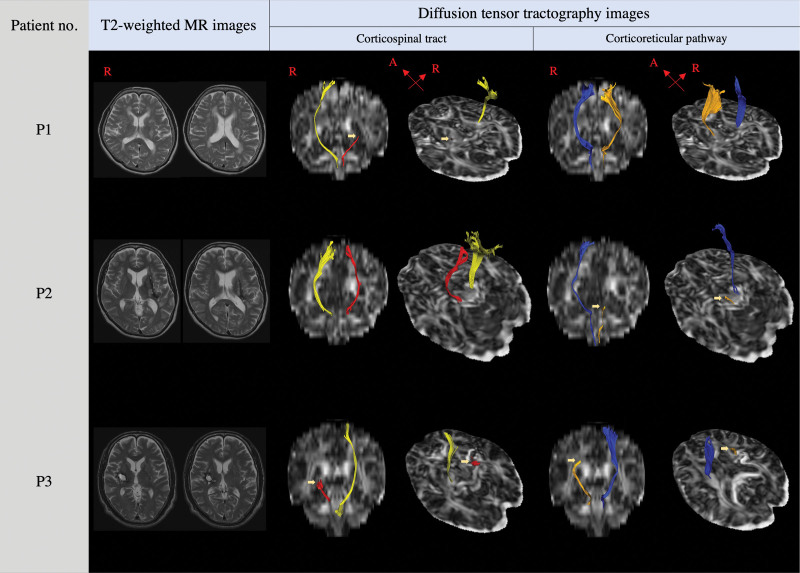
T2-weighted brain MR images (A) and diffusion tensor tractography image (B) of the CST and CRP for the 3 patients (*arrow:* discontinuation of a neural tract). CRP = corticoreticular pathway, CST = corticospinal tract.

In P1, the CST was discontinuous at the basal ganglia level, and the CRP originated from the supplementary motor area and descended through the known CRP pathway (group B, Fig. 1). In P2, the CRP was discontinuous at the midbrain, and the CST originated from the primary motor area and descended through the known CST pathway (group C, Fig. 1). Both the CST and the CRP of P3 were discontinuous in (group D, Fig. 1).

### 2.2. Clinical evaluations

The patients’ clinical evaluations were performed at the time of admission, 1 and 2 months after admission, and at the time of discharge. Each patient was assessed using the Fugl-Meyer assessment (FMA) to measure the severity of motor paralysis and sensory disturbances. The lower-extremity motor subscale of the FMA (FMA-LE) was used to determine the patients’ FMA motor score. The modified Ashworth Scale was used to evaluate the muscle spasticity of the ankle plantar flexor muscle, and the muscle strength on both the affected and unaffected sides was assessed for knee joint extension. The patients’ performance was assessed using the Trunk Impairment Scale (TIS), the Mini-Balance Evaluation Systems Test (Mini-BESTest), and the Functional Ambulation Categories (FAC). Walking speed was measured by a stopwatch marking when the patient passed the start and end lines of a 10-m walkway.

In P1, some extent of recovery was observed regarding the weakness of her right lower extremity (from the FMA-LE score of 15–23, cutoff point: 21^[16]^) and trunk function (from the TIS score of 10–20, cutoff: 12^[17]^) over a 2-months period following her admission. The improvement in her balance performance stalled (from the Mini-BESTest score of 4–15, cutoff: 17.5^[18]^), but she was able to walk independently on an even floor at discharge (FAC score improvement from 0–4). However, her gait speed was markedly lower than the cutoff point 0.49 m/s in a previous study,^[14]^ and her gait speed deteriorated from 0.22–0.19 m/s (Table 2).

**Table 2:** Changes in the patients’ clinical data.

Patient no.		Ad-mission	1 month	2 months	Discharge
P1	FMA-LE, 0–34	15	21	23	23
	FMA sensory score, 0–12	6	6	6	6
	MAS, 0–5	0	0	0	0
	Knee extensor strength, affected side, Nm/kg	0	0.78	0.86	0.87
	Knee extensor strength, unaffected side, N/kg	0.77	1.32	1.36	1.52
	TIS, 0–23	10	16	18	20
	Mini-BESTest, 0–28	4	5	13	15
	FAC, 0–5	0	2	2	4
	Maximum gait speed, m/s	−	0.22	0.21	0.19
	Step length symmetry magnitude, ratio	−	8.54	1.08	1.19
	Swing time symmetry magnitude, ratio	−	1.60	1.32	1.05
P2	FMA-LE, 0–34	21	23	23	25
	FMA sensory score, 0–12	6	6	6	6
	MAS, 0–5	1^+^	1^+^	1^+^	1^+^
	Knee extensor strength, affected side, Nm/kg	0.79	1.95	1.97	1.99
	Knee extensor strength, unaffected side, N/kg	1.08	2.16	2.16	2.20
	TIS, 0–23	17	19	22	23
	Mini-BESTest, 0–28	18	24	26	26
	FAC, 0–5	2	3	4	5
	Maximum gait speed, m/s	0.37	0.54	0.8	1.01
	Step length symmetry magnitude, ratio	0.98	0.75	0.90	1.07
	Swing time symmetry magnitude, ratio	1.18	1.36	1.12	1.24
P3	FMA-LE, 0–34	11	19	19	19
	FMA sensory score, 0–12	4	4	6	6
	MAS, 0–5	0	0	1	1
	Knee extensor strength, affected side, Nm/kg	0.80	0.93	0.93	1.06
	Knee extensor strength, unaffected side, N/kg	1.88	2.86	3.26	3.52
	TIS, 0–23	13	15	17	18
	Mini-BESTest, 0–28	3	7	16	18
	FAC, 0–5	1	3	3	4
	Maximum gait speed, m/s	0.17	0.24	0.47	0.46
	Step length symmetry magnitude, ratio	15.65	1.03	1.25	1.54
	Swing time symmetry magnitude, ratio	2.39	1.51	1.67	1.67

FAC = Functional Ambulation Categories, FMA sensory score = sensory score of the Fugl-Meyer assessment, FMA-LE = lower-extremity motor subscale of the Fugl-Meyer assessment, MAS = Modified Ashworth Scale, Mini-BESTest = Mini-Balance Evaluation Systems Test, TIS = Trunk Impairment Scale.

P2 showed the best course of the 3 patients. The weakness of his right lower extremity improved (from the FMA-LE score of 21–25, cutoff: 21^[16]^), as did his trunk function (TIS score from 17–23, cutoff: 12^[17]^) and balance performance (Mini-BESTest score from 18–26, cutoff: 17.5^[18]^). Consequently, he was able to walk independently at discharge, as his FAC score had improved from 2 to 5. The improvement in his gait speed was also excellent (from the gait speed of 0.37–1.01 m/s, cutoff: 0.49 m/s^[19]^) (Table 2).

In P3, because he was classified in group D, his walking ability was expected to have a poor prognosis.^[10]^ Contrary to expectations, the improvement in the weakness of his left lower extremity stalled (FMA-LE score from 11–19, cutoff: 21^[16]^), but he achieved a degree of recovery of his trunk function (TIS score from 13–18, cutoff: 12^[17]^) and balance performance (Mini-BESTest score from 3–18, cutoff: 17.5^[18]^). His gait speed improved to around the cutoff point (from the gait speed of 0.17–0.46 m/s, cutoff: 0.49 m/s^[19]^), and he was able to walk independently on an even floor at discharge, as his FAC score rose from 1 to 4 (Table 2).

### 2.3. Three-dimensional gait analysis

A three-dimensional (3D) gait analysis was performed in each patient at the time of admission, 1 and 2 months after admission, and at the time of discharge using a 3D motion analysis system (Kinema Tracer, Kissei Comtec, Tokyo, Japan) to determine abnormal gait patterns and spatiotemporal gait asymmetry. All measurement protocols followed the procedure of described for gait measurement in hemiplegic stroke patients.^[20,21]^ The abnormal gait patterns identified were circumduction, hip hiking, lateral shift of the trunk, forefoot contact, extensor knee thrust, retropulsion of the hip, knee flexed gait, contralateral vaulting, insufficient knee flexion during swing phase, and medial whip by following the method of Mukaino.^[22]^ We calculated the index value of each abnormal gait pattern from the 3D coordinates’ axis data (X, Y, and Z coordinates indicate latero-medial, anteroposterior, and vertical data, respectively), and we calculated the deviation value using the healthy subject data^[20,21]^ and the following equation^[22]^: T = 50 + 10 × (X − μ)/δ, T represents the deviation score, X is the individual’s value, μ is the mean raw value of the healthy subjects, and δ is the standard deviation. Radar charts of abnormal gait indices were created from the calculated deviation values of the 10 types of abnormal gait patterns and then visually inspected.^[22]^

The average score of the healthy subjects was set to 50, and the normal range of each index value was the average score of the healthy subjects ± 2 SD.^[22]^ A higher score indicated a greater abnormality.^[22]^ Regarding the spatiotemporal gait asymmetry, we used the values for left and right swing times and step lengths obtained from the 3D gait analysis. The magnitudes of the swing time and step length asymmetries were calculated as the ratio of the left and right values, with the larger value in the numerator (irrespective of the affected side) as described.^[23]^ A ratio of 1.0 indicates perfect symmetry.

P1 showed an abnormal gait pattern at 1 month, including extensor knee thrust, retropulsion of the hip, contralateral vaulting, insufficient knee flexion, and hip hiking. At discharge, the retropulsion of the hip had improved, but the extensor knee thrust, contralateral vaulting, insufficient knee flexion, and hip hiking remained. Among them, knee sway during the stance phase due to the extensor knee thrust was remarkable. In addition, spatiotemporal gait asymmetry was observed in P1 at her admission, but improved over the minimal clinically important difference level and the patients developed symmetrical gait during the follow-up.

In P2, the abnormal gait pattern was milder than in the other cases. Compared to admission, the retropulsion of the hip was improved at discharge, but the insufficient knee flexion and lateral shift of the trunk remained at discharge. In other words, the compensatory pattern caused by the lateral shift of the trunk against insufficient knee flexion was remarkable. In P2’s gait asymmetry, only temporal gait asymmetry was observed at admission, but it improved to symmetry at discharge.

**Figure 2.**
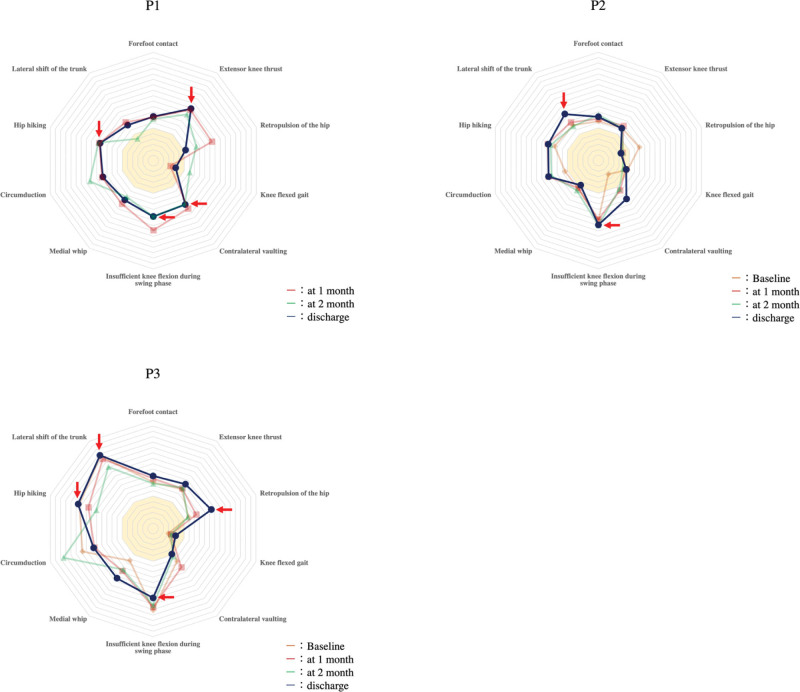
Radar chart of abnormal gait indices. The average score of healthy subjects is set to 50. A high standard score represents high abnormality.

P3’s gait pattern was more markedly abnormal than those of P1 and P2. Retropulsion of the hip, insufficient knee flexion, hip hiking, and lateral shift of the trunk were observed from admission to discharge. The compensatory pattern against insufficient knee flexion (i.e., hip hiking and lateral shift of the trunk) was particularly remarkable. Spatiotemporal asymmetry was also present from admission to discharge (Table 2; Fig. 2).

## 3. Discussion

This case series study is the first report of an analysis of the characteristics of gait disturbance based on CST and CRP injury types. Our analyses revealed that a certain degree of gait is possible regardless of the CST and CRP injury types (i.e., independent level ground gait). However, the gait pattern clearly differed based on the types of injury, particularly in the patient with injuries of both the CST and CRP that resulted in a markedly residual abnormal gait pattern.

In P1, the CST was discontinuous, but the CRP was intact. The CRP innervates trunk and proximal muscles and contributes to predictive postural control and gait.^[11]^ In their mini-review, Jang and Lee^[24]^ pointed out that the degree of CRP injury in post-stroke patients affects gait performance. Although the CST is an important prognostic indicator for estimating the prognosis of gait independence, even patients with an injured CST have been able to achieve gait independence.^[25]^ In the case of P1, the intact CRP might ensure the gait performance and eventually led to gait independence. In contrast, P1 showed a marked decrease in gait speed. The gait speed in post-stroke patients is affected by motor function in the affected lower limb and muscle strength in the unaffected lower limb.^[26,27]^ Compared to the other 2 patients, P1 had decreased knee extensor strength on the affected and unaffected sides, and these factors could have influenced the decrease in gait speed.

In P2, the CRP was discontinuous, but the CST was intact. The CST innervates distal limb muscles and is widely regarded as a predictive indicator in the recovery of motor function and gait performance in post-stroke patients.^[28]^ In fact, P2 had excellent physical function, including motor function in affected lower limb, trunk function, and balance function, and he eventually achieved gait independence. His clinical course supports previous studies’ findings and suggests that a high level of gait performance can be achieved if the CST is intact.

In P3, both the CST and CRP were discontinuous. An earlier investigation revealed that patients with injury to both the CST and CRP had a significantly reduced level of gait independence.^[10]^ However, contrary to our expectations, P3’s gait performance improved to gait independence. Age is an important predictor of gait independence, and younger post-stroke patients are more likely to achieve gait independence regardless of the neurological status.^[25,29]^ The muscle strength in the unaffected lower limb is also important for gait performance.^[30]^ In fact, compared to the other patients, P3 had greater knee extensor strength on the unaffected sides. Despite the injury to both the CST and CRP in P3, his age and muscle strength in the unaffected lower limb could have had a positive influence, leading to gait independence.

Interestingly, although all 3 patients improved to the level of gait independence, their gait patterns differed. The gait pattern of P1 showed extensor knee thrust and insufficient knee flexion due to lower-limb dysfunction caused by motor paralysis (Fig. 2). An individual with a complete CST injury exhibited a markedly abnormal gait pattern compared to patients with partial CST injury.^[31]^ Since the CST innervates distal limb muscles,^[11]^ P1 (with complete CST injury) could impair lower-limb stability and swing motion of her lower limb due to motor paralysis. P2 showed abnormalities in insufficient knee flexion and lateral shift of the trunk (Fig. 2), and of these, the lateral shift of the trunk is a specific gait pattern that was not observed in P1. This gait pattern is a compensatory movement to complement the forward translational movement of the paralyzed leg during the swing phase,^[32]^ and it often results from weakness of hip flexors. Because the CRP innervates the trunk and proximal muscles of the lower extremities and is involved in the neuromuscular control of gait,^[11]^ this compensatory behavior might be a motor abnormality caused by damage to CRP.

P3, with both the CST and CRP injured, showed a generally strong abnormal gait pattern. Among the 3 patients, the gait patterns caused by motor paralysis and compensatory movements to supplement forward translational movements (such as insufficient knee flexion, hip hiking, and the lateral shift of the trunk) were mixed. As noted above, the CST and CRP have distinct roles, and the modulation of gait control is more pronounced in the case of injury to both the CST and CRP.^[33]^ We speculate that although P3 achieved gait independence due to age and muscle strength on the unaffected side, the neurological factors resulted in the more abnormal gait pattern that remained.

This case series study suggests that even if injured both the CST and CRP are injured, a degree of gait is possible, depending on factors such as age and knee extensor strength on the unaffected side. However, the injury of both the CST and CRP was observed to result in a markedly abnormal gait pattern, and thus intervention to correct the gait pattern may be necessary for such patients from an early post-onset time point.

This study has several limitations to consider. Since this was a case series study of only 3 patients, the generalizability of our findings to all patients with stroke is limited. We have plans for research studies with a large sample size. In our DTI analysis, we did not consider the other neural pathways known to be involved in gait, such as the vestibulospinal tract and rubrospinal tract.^[32]^ Further investigations that include analysis of these tracts are necessary. The patients’ DTI data were obtained only at the time of admission; because temporal changes in the fiber tracts are involved in the recovery of gait performance, longitudinal evaluations of DTI data may reveal more detailed subtypes. Lastly, DTI analyses might underestimate the fiber tracts because of the effects of hematoma and edema. For compensation of this DTI limitation, a combined study with transcranial magnetic stimulation would be worthwhile.

## Author contributions

**Conceptualization:** Ren Fujii, Makoto Tamari, Naomichi Mizuta, Naruhito Hasui, Yuki Nonaka.

**Data curation:** Ren Fujii, Yuki Nonaka.

**Formal analysis:** Ren Fujii.

**Investigation:** Ren Fujii, Fumiaki Tamiya, Misato Horinouchi, Hiroshi Hosokawa.

**Methodology:** Ren Fujii, Makoto Tamari, Naomichi Mizuta, Naruhito Hasui, Yuki Nonaka.

**Project administration:** Ren Fujii.

**Supervision:** Hiroshi Hosokawa, Shinichiro Tanaka.

**Visualization:** Ren Fujii.

**Writing – original draft:** Ren Fujii.

**Writing – review & editing:** Ren Fujii, Makoto Tamari.
